# Static posturography in aging and Parkinson's disease

**DOI:** 10.3389/fnagi.2012.00020

**Published:** 2012-08-06

**Authors:** Guntram W. Ickenstein, Helmut Ambach, Antonia Klöditz, Horst Koch, Stefan Isenmann, Heinz Reichmann, Tjalf Ziemssen

**Affiliations:** ^1^Department of Neurology and Stroke Unit, HELIOS General Hospital Aue, University of DresdenDresden, Germany; ^2^Department of Neurology, Carl Gustav Carus University Hospital, University of DresdenDresden, Germany; ^3^Institute of Biostatistics, HELIOS General Hospital Aue, University of DresdenDresden, Germany; ^4^Department of Neurology and Neurophysiology, HELIOS General Hospital Wuppertal, Witten-Herdecke UniversityWuppertal, Germany

**Keywords:** Parkinson's disease, aging, posturography, balance testing, Romberg-test

## Abstract

**Introduction:** In clinical practice, evaluation of postural control is based on the neurological examination, including Romberg's test, examination of gait and performance of pull test as part of the Unified Parkinson's Disease Rating Scale (UPDRS). The goal of our study was to identify posturographic parameters since quantitative technical methods for the measurement of postural control are not established in clinical routine yet. **Methods:** In this cross-sectional study design we examined patients with Parkinson's disease (PD) (Hoehn and Yahr < 3; PD *n* = 12) on a static posturographic platform (eyes open and eyes closed), performing a standard Romberg's test during neurological examination and compared the results with an age-matched healthy adult control (HAC *n* = 10) and a healthy young control (HYC *n* = 21). **Results:** In the platform Romberg's test with open eyes, the patients with PD showed a significantly greater mean sway [PD: 14.98 vs. HAC: 8.77 (mm), *p* < 0.003 vs. HYC 7.80 (mm)], greater mean radius [PD: 28.31 vs. HAC: 16.36 (mm), *p* < 0.008 vs. HYC: 14.19 (mm)] and greater marked area [PD: 2.38 vs. HAC: 0.88 (cm^2^), *p* < 0.016 vs. HYC: 0.78 (cm^2^)] compared to the HAC. The Romberg's test with closed eyes revealed a significantly greater mean sway [PD: 13.83 vs. HAC: 10.12 (mm), *p* < 0.033 vs. HYC: 5.82 (mm)] and greater mean radius [PD: 25.03 vs. HAC: 18.15 (mm), *p* < 0.045 vs. HYC: 9.11 (mm)] compared to both groups. **Conclusions:** The platform Romberg-test with closed eyes detected significant differences in elderly people and patients with Parkinson's disease, which could be objectively quantified with static posturography testing. Age alone showed significant changes, only detectable with closed eyes. Therefore, balance testing with a new computerized approach could help to identify balance problems in a geriatric assessment in clinical routine, especially with the parameters marked area and mean sway.

## Introduction

Good balance is an imperative skill for daily life that requires the complex integration of sensory information regarding the position of the body relative to the surroundings and the ability to generate appropriate motor responses to control body movement. Information from sensory systems concerning the environment is used to influence the balance and coordination (Whipple et al., 1993; Brooke-Wavell et al., 2003). In this way motor reactions are triggered in advance so that balance is maintained. Studies in elderly people found poor postural control in relation to a reduction in visual information (Judge et al., 1995; Liaw et al., 2009; Palm et al., 2009). The most important roles of the equilibrium system are the triggering of reflexes to maintain balance and the adjustment of head and eyes despite head and body movements. Furthermore, it provides possible spatial orientation, registers the body's movements and stabilizes upright posture (Sturnieks et al., 2008). With aging the systems involved experience a progressive decline of their physiological functions that appear as a multi-factor occurrence with various components and can contribute to balance deficits. Balance disorders represent a growing public health concern due to the association with falls and fall-related injuries. Balance problems and falls can mark the beginning of a decline in independence and are the leading cause of injury-related hospitalization in older people. In studies one in three people over the age of 65 years who are living in the community experience at least one fall each year and 10–15% of these falls are associated with serious injury. Economically the direct and indirect costs associated with falls are large and will grow as the proportion of older people increases. Therefore, it is important to detect balance problems in older people and reduce the injury-related burden on individuals and society (Horak et al., 1989; Horak, 2006). In clinical practice, evaluation of postural control is based on the neurological examination, including Romberg-test, examination of gait and performance of pull test. Quantitative and technical methods for the measurement of postural control in the geriatric assessment are not established for clinical routine yet. Especially in patients with Parkinson's disease (PD) the primary symptoms include the dysfunction of the postural reflexes. It is possible that lesions in non-dopaminergic systems contribute to the pathophysiology of postural instability in PD (Chaudhuri et al., 2006; Truong et al., 2008). Recent studies show that the gait-related hyperactivity of the mesencephalic locomotor region correlates with clinical parameters (gait severity and disease duration), but not with the degree of mesencephalic atrophy. Freezing of gait might emerge when altered cortical control of gait is combined with a limited ability of the mesencephalic locomotor region to react to that alteration (Ziemssen and Reichmann, 2007; Grimbergen et al., 2009). These limitations might become particularly evident during challenging events and could be tested in a technical approach in a standardized platform balance test.

**Figure 1:**
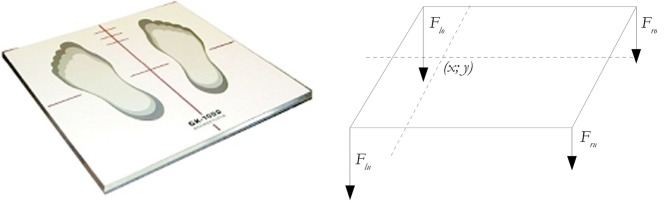
**The static posturography platform (GK-1000) is a computerized coordination and balance analysis system.** With the help of four piezoelectric power sensors, the position and movement of the projection of the center of gravity can be measured and tracked.

Current management of balance problems is hampered by the subjective and variable nature of the available clinical balance measures. Electronic posturography studies with the help of new software tools offer several advantages and could provide a useful tool to gain a better understanding of the pathophysiological mechanisms in patients with balance disorders (Visser et al., 2008; Snijders et al., 2011; Zwergal et al., 2011). Since static posturography can nowadays be measured and analyzed with specialized techniques, it is possible to further evaluate the applicability of static posturography in clinical routine (see Figure 1). The aim of this study was to establish a technical method that would show significant differences in balance in elderly people and those with PD. The null hypothesis of our study is that a balance platform test would show no difference between a healthy young control (HYC) and a healthy adult control (HAC). In addition no significant differences would be detectable between a healthy age-matched adult control and patients with PD.

## Methods

Patients with PD were tested bare foot and in the morning hours between 9:00 and 10:00 o'clock, after taken their regular medication. The healthy age-matched control group was made up of volunteers (relatives/family members) without any neurological diseases. The HYC served as a control group to measure the aging effects. A standardized posturographic test and examination procedure was established to reduce variability. Patients with PD were examined during an outpatient visit, assessing the clinical scores [Hoehn and Yahr, Unified Parkinson's Disease Rating Scale (UPDRS), and Schwab and England Scale] including a pull test. Before inclusion of a patient in the study a neurological examination was performed in all subjects, to exclude proprioceptive function problems (e.g., orthopedic problems and visual problems), signs of subcortical arteriosclerotic encephalopathy with neuroimaging and health-related sociological or psychological factors that could compromise compliance with the study protocol. Afterwards the responsible physician carried out an evaluation with regard to the inclusion criteria of the investigation protocol authorized by the Ethics Commission of the University of Dresden (EK-Nr.:2531022007) in accordance with good clinical practice (GCP) guidelines. Inclusion criteria were: (1) Patients diagnosed with PD, Hoehn and Yahr stage <3, (2) No comorbidities that may affect gait balance including proprioceptive function, (3) Written consent. The statistical analysis was carried out with SPSS 17.0 (SPSS Inc.) and Excel 2007 (Microsoft Corp.). The normal distribution of data was confirmed with appropriate histograms. In case of variance equality, comparisons between the groups (PD, HAC, and HYC) were made with the unpaired *t*-test for random samples. With variance inequality Welch's *t*-test for unpaired random samples was used. To evaluate mean value differences between the groups, a univariate variance analysis ANOVA was carried out (*post-hoc* analysis according to Bonferroni). Highly significant results were those with a significance level of *p* < 0.000. Significant results showed a significance level of *p* < 0.05. The relationships were graphically represented in the form of integrated Box Whisker plots (Ladislao and Fioretti, 2007).

**Figure 2:**
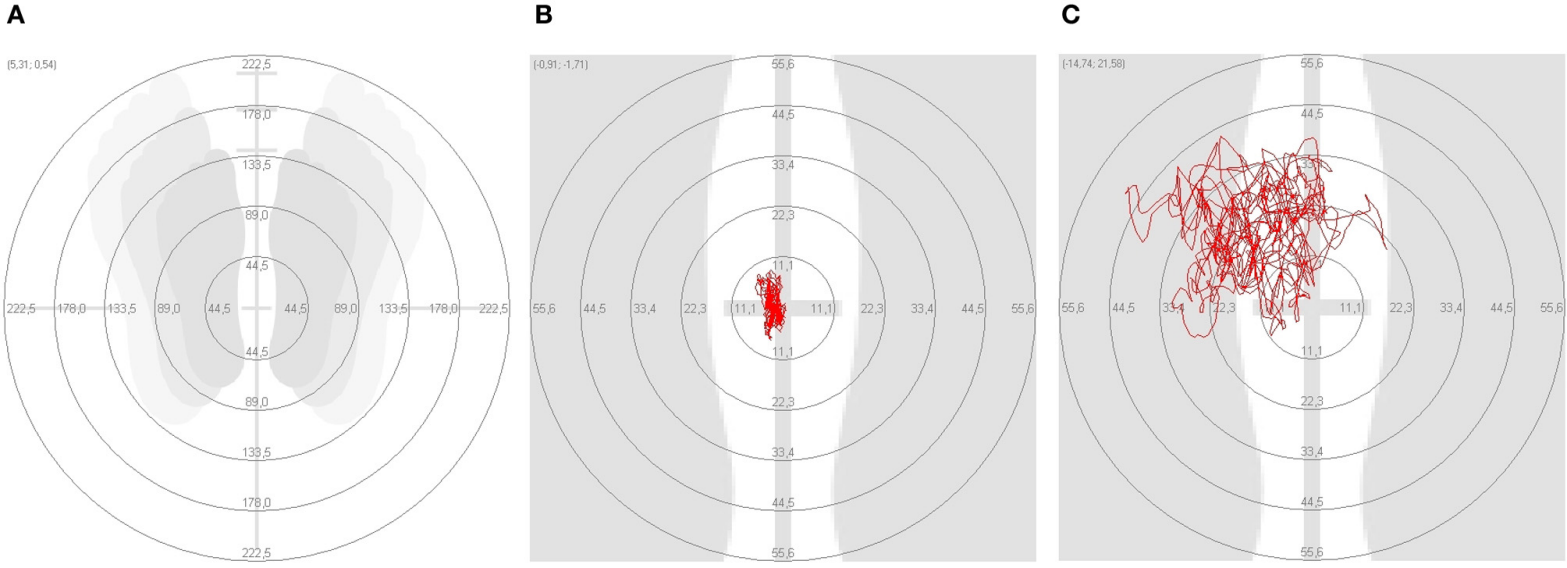
**On the platform test the different posturographic parameters were compared. (A)** Standing position; **(B)** marked area in a healthy adult control (HAC); **(C)** marked area in a patient with Parkinson's disease.

All subjects (PD, HAC, and HYC) were placed upright on the posturography platform with horizontally raised arms (Romberg's test) (see Figure 2). Each individual was measured twice to reproduce the results. Double measurements were made regarding the subject's balance during a modified Romberg standing test (track width = 10 cm) on the balance platform, first with open, then with closed eyes. The position of the arms, which diverges from the usual procedure for posturographic measurements, serves as a provocation mechanism (extension of the arms horizontally forward and turn of the palms upward). The extension of the arms causes a forward shift in the center of gravity, and also provides mental distraction. Before starting the measurement, the participants were asked to stand as still as possible. Verbal instruction to the subjects should adhere to the same text, as much as possible. If the participant's central pressure point (red point) is located on the outer edge of the measurement field, the patient's position can be adjusted. After 30 s a signal sounds and the participant can lower his arms and rest. Afterwards the second measurement is performed with closed eyes (Zok et al., 2008).

With the electronic registration of the subject's balance regulation, the x and y coordinates of the center of gravity can be determined with the help of electronic amplification. This point shows the oscillation of the body during the Romberg's test on the platform (Nebel, 2005). The following parameters were calculated according to the study protocol:

1. ***The mean radius*** indicates the average distance of all measurements from the center point (zero-point), measured in meters. The parameter angle reports the direction to which the subject corrects her/his balance most often. The center point of all measurement values is the center point of all x/y coordinates of the measurement.
2. ***The mean sway*** represents the average distance of all measurements from the center of all measurements (measured in meters).
3. ***The marked area*** is the surface that is described during the measurement of the center of gravity of the subject. The calculation of the surface is carried out graphically with a resolution of 0.0025 cm^2^. Continuous triangles from the mean value of all measurement values to the last measurement point to the current measurement point are calculated. Points on the grid which overlap numerous times are not counted more than once (measured in square meters).
4. ***The mean speed*** of the central pressure point is a projection of the subjects' center of gravity on the measurement platform. It is the speed at which the central pressure point of the subjects moves on the platform and is measured in meters per second.

## Results

In this cross-sectional study, patients with PD, HACs and HYCs were included (see Table 1).

**Table 1:** **Descriptive parameters of the study population**.

**Group**	**Percentage of females**	**Mean age (SD)**	**Percentage of regular exercise**	**Mean height (SD)**	**Mean weight (SD)**
Healthy young control (HYC, *n* = 21)	71% (15/21)	22.4 (4.6)	86% (18/21)	168.3 (3.4)	69.7 (7.4)
Healthy adult control (HAC, *n* = 10)	60% (6/10)	72.0 (6.9)	70% (7/10)	165.0 (4.5)	73.3 (5.8)
Patients with Parkinson's disease (PD, *n* = 12)	50% (6/12)	71.9 (5.8)	83% (10/12)	167.5 (3.8)	80.9 (6.7)

The neurological status of the participants was assessed according to the modified scale by Hoehn und Yahr and assigned to one of the various stages (PD stage < 3 vs. HAC stage 0 vs. HYC stage 0). In addition the Schwab and England scale (PD 81% vs. HAC 100% vs. HYC 100%) and the pull test (PD 1.17 vs. HAC 0 vs. HYC 0) were used. The cross-sectional comparison showed no statistical difference in age between patients with PD and the HAC.

In the platform Romberg-test **with open eyes** patients with PD showed a significantly greater mean sway [PD: 14.98 vs. HAC: 8.77 vs. HYC: 7.80 (mm)] with HYC vs. HAC *p* = 0.67, HYC vs. PD *p* = 0.0005, and PD vs. HAC *p* = 0.003. In addition a greater mean radius [PD: 28.31 vs. HAC: 16.36 vs. HYC: 14.19 (mm)] with HYC vs. HAC *p* = 0.66, HYC vs. PD *p* = 0.0009, and PD vs. HAC *p* = 0.008 and greater marked area [PD: 2.38 vs. HAC 0.88 vs. HYC: 0.78 (cm^2^)] with HYC vs. HAC *p* = 0.35, HYC vs. PD *p* = 0.002, and PD vs. HAC *p* = 0.016 could be found. A significant difference of mean speed [PD: 20.13 vs. HAC: 13.01 vs. HYC: 9.77 (mm/s)] with HYC vs. HAC *p* = 0.07, HYC vs. PD *p* = 0.0005, and PD vs. HAC *p* = 0.177 could be detected between patients with PD and the HYC. However, no significant difference was seen between the HAC and patients with PD.

**Figure 3:**
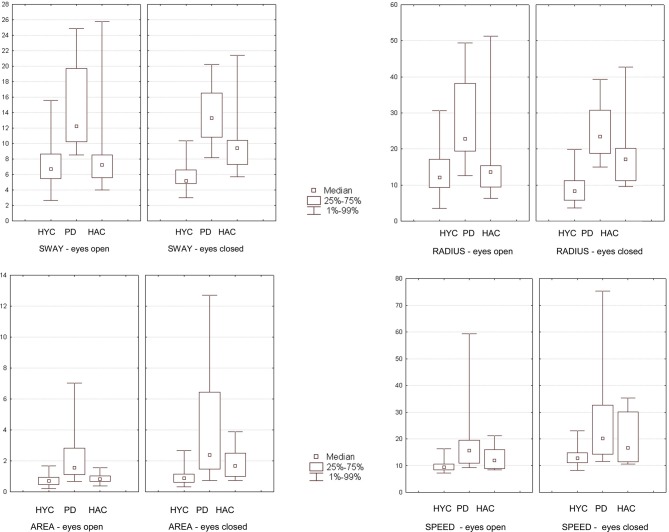
**The results of the group comparison in the Romberg-test with eyes open and eyes closed are presented graphically as box plots (HYC = healthy young control; PD = patients with Parkinson's disease; HAC = healthy adult control).** Statistical significant findings are seen especially with closed eyes.

Especially in the Romberg-test **with closed eyes** most parameters of the study examination showed highly significant differences: greater mean sway (with HYC vs. HAC *p* = 0.0007, HYC vs. PD *p* = 0.000009, and PD vs. HAC *p* = 0.033); greater mean radius (with HYC vs. HAC *p* = 0.002, HYC vs. PD *p* = 0.000009, and PD vs. HAC *p* = 0.045); greater marked area (with HYC vs. HAC *p* = 0.010, HYC vs. PD *p* = 0.0005, and PD vs. HAC *p* = 0.211) and greater mean speed (with HYC vs. HAC *p* = 0.104, HYC vs. PD *p* = 0.002, and PD vs. HAC *p* = 0.249). However, the examination with open eyes revealed no significant differences comparing HYC vs. HAC. Therefore, aging effects could only be detected with closed eyes comparing HYC with HAC (see Figure 3).

## Discussion

A multi-modal system of information collection and processing involves the vestibular, kinaesthetic, visual, and tactile analyzer and is required for the overall coordination of balance. Locomotion in humans and other vertebrates is based on spinal pattern generators, which are regulated by supraspinal control. The activation processes include the interfastigial cerebellum and bilateral midbrain tegmentum (cerebellar and mesencephalic locomotor regions), their descending target regions in the pontine reticular formation, and the rhythm generators in the cerebellar vermis and paravermal cerebellar cortex (Jahn et al., 2008; Schapira, 2008; Siderowf and Stern, 2008). For a clinical routine assessment of balance problems a robust technical method is still needed. One goal of our study was the evaluation of a technical approach with a balance platform test using new techniques and software. Significant differences were detected between a HYC and a HAC only when eyes were closed with mean sway (*p* = 0.0007), mean radius (*p* = 0.002), and marked area (*p* = 0.010). Postural control during standing involves a low intensity cerebellar activity and sensorimotor control via the thalamus and basal ganglia. During aging the vestibular system shows declines with loss of hair cells and ganglion cells, but the vestibulo-ocular reflex (VOR) is not affected so early (Jahn et al., 2004). The results confirm observations which described poor postural control in relation to a reduction in sensory information in older subjects. Whipple et al. found poor balance in older subjects on a dynamic posturography platform both when they were given inaccurate visual information and when they closed their eyes (Whipple et al., 1993). In addition Liaw et al. indicate a decreased stability in the elderly, especially when subjects stood on an unstable platform and when visual information was limited or contradictory (Liaw et al., 2009). Comparing these results it can be concluded that if an important system is impaired (for example the visual system when eyes are closed) and the physiological compensation mechanisms are overwhelmed, it is possible to posturographically detect a worsening in postural balance (Judge et al., 1995; Palm et al., 2009). In clinical practice the pull test allows an assessment of the motor reaction to a sudden backwards pull on the shoulders so far. But the repeatability of the pull test is limited by the varying sizes and weights of the patient and clinician. Some examiners warn the patient before performing the shoulder pull, others do not. A unified standard equipment-based examination for the quantitative assessment of postural stability is important and should be established in the clinical setting. Our results show that aging effects are detectable even in a small sample size and that balance problems in aging can be best observed with closed eyes assessing the marked area, mean radius, or mean sway on a balance platform test.

In addition our study found highly significant and robust results comparing a HAC and patients with PD. So far studies on patients with PD could not detect significant differences on a balance platform test but found that among the variables determined to be relevant, the presence of subcortical, arteriosclerotic encephalopathy was a significant factor for increased sway (Ebersbach et al., 2002). The authors concluded that coinciding cerebral micoangiopathy leads to more serious balance impairment in idiopathic PD. The question arises as to whether cerebrovascular morbidity must coincide in order to result in significant changes in the posturographic measurements. In our study only patients without signs of subcortical, arteriosclerotic encephalopathy or cerebrovascular disease were included, and therefore the presence of cerebrovascular comorbidity was purposefully excluded. Also in contrast to the standard procedure in other studies, our participants were asked to extend their arms horizontally forward and to turn their palms upward. The extension of the arms causes a forward shift in the center of gravity, and also provides mental distraction. This “dual task” is accepted as a provocation method like others such as the use of a foam rubber pad or a dynamic shifting of the measurement platform (Rossi et al., 2009). The results obtained between the patients with PD and the HAC showed robust and significant differences in a standardized platform test with selected technical parameters. Our study has several limitations which must be considered when interpreting these results. Our small study sample was restricted to individuals with idiopathic PD and exclusion of co-morbidities, and thus is not representative of Parkinson patients in general. We emphasize that the selection of subjects from a single hospital may restrict the degree to which our findings may be generalized to other regions. The strength of our study is a well selected group to allow a test in a highly homogonous small sample size. In terms of the four posturographic parameters analyzed (radius, sway, area, and speed) the groups differ significantly in marked area, mean radius and mean sway with open eyes as well as mean radius and mean sway with closed eyes. In summary it seems that mean sway is the best parameter to be assessed in patients with PD. Grimbergen et al. hypothesize that cell loss in the locus coeruleus and a resultant central norepinephrine deficit are intimately involved in the pathophysiology of postural instability in PD and new treatment options would aim to correct postural instability and preventing falls (Ziemssen and Reichmann, 2007; Grimbergen et al., 2009). Interestingly mental imagery of standing during fMRI revealed a reduced activation of the mesencephalic brainstem tegmentum and the thalamus in patients with postural imbalance and the authors conclude that imbalance and falls in progressive supranuclear palsy (PSP) are closely associated with thalamic dysfunction (Jahn et al., 2004). However, deficits in thalamic postural control get most evident when balance is assessed during modified sensory input. Therefore studies conclude that reduced thalamic activation via the ascending brainstem projections may cause postural imbalance (Rossi et al., 2009; Zwergal et al., 2011). Deutschländer et al. showed that blind subjects rely more on vestibular and somatosensory feedback for locomotion control than sighted subjects. This is accompanied by enhanced voluntary motor control and enhanced motor-kinesthetic processing. Therefore, locomotor training most likely appears to change the excitability of simple reflex pathways as well as more complex circuitry and can be used for geriatric and neurological rehabilitation (Rossignol, 2006; Deutschländer et al., 2009).

Our posturographic examinations were carried out on medicated patients, independent of the dosing regime of dopaminergic substances. Beuter et al. describe a mildly positive effect of L-Dopa on postural control, but it must be assumed that posturographic instability is one of the symptoms of PD that does not respond well to L-Dopa, and no significant improvement can be achieved by dopaminergic substitution (Beuter et al., 2008). In comparison the posturographic performance of patients with balance problems can be favorably influenced by appropriate balance training (Hirsch et al., 2003). Hirsch et al. studied the effects of two exercise programs for improving postural control. Nine patients participated in balance training and six in a combination of balance and strength training. All the patients participated for 10 weeks, three times per week on non-sequential days. Their balance was evaluated by means of dynamic posturography before and after the 10-week training program, and again 4 weeks after the program had ended. Both groups showed improved balance directly after the training period. After 4 weeks, however, only the group who had completed the combined training showed a lasting effect (Hirsch et al., 2003). Since the fear of falling limits daily activities in elderly individuals, the evaluation of the postural stability or instability should be carried out more often in the clinical practice to support a balance training program (Mitchell et al., 1995; Balash et al., 2005; Błaszczyk et al., 2007). The aim of this study was to find robust parameters for posturographic measures and evaluate this method in order to find objective parameters that will determine balance problems. The differences shown are robust in a small study population even with only some of the four parameters that are consistently significant throughout the different group comparisons. Our study results are therefore too preliminary in order to propose this method as a regular clinical practice test to asses balance problems. Especially our method may still lack applicability since it was not confronted with other methods that directly measures balance status and there are numbers of risk factors which produce temporary balance disturbances, e.g., medications, dizziness, low blood pressure, etc., and these influences can not necessary be picked up by this method. In the future posturographic studies should address these limitations and could also focus on therapeutic effects to evaluate interventions aimed to increase balance.

## Conclusion

The Romberg Test combined with a provocation method (dual task) and a technical approach on the posturographic platform could be suitable for the routine evaluation of balance problems in order to initiate the appropriate therapeutic measures for the improvement of postural control. Since nowadays software packages can determine various parameters characterizing balance problems, the results are more comparable. However, static posturography should also be supplemented by additional scores, for example the Functional Reach Test or the Pull Test and Lean Forward Test. Furthermore, future studies on balance could use advanced neuroimaging and neurochemical techniques to analyze detailed clinical balance. In our study the platform Romberg-test showed significant group dependent results especially with closed eyes, reflecting differences in postural stability. Therefore, we highly recommend the use of a standard platform measurement test in a geriatric setting. Especially the marked area and mean sway seem to be reliable parameters for monitoring balance in clinical practice.

### Conflict of interest statement

The authors declare that the research was conducted in the absence of any commercial or financial relationships that could be construed as a potential conflict of interest.
